# Exploratory analysis of the effect of a dexamethasone-sparing regimen for prophylaxis of cisplatin-induced emesis on food intake (LUNG-NEPA study)

**DOI:** 10.1038/s41598-023-28464-9

**Published:** 2023-01-23

**Authors:** Luigi Celio, Diego Cortinovis, Alessio Aligi Cogoni, Luigi Cavanna, Olga Martelli, Simona Carnio, Elena Collovà, Federica Bertolini, Fausto Petrelli, Alessandra Cassano, Rita Chiari, Francesca Zanelli, Salvatore Pisconti, Isabella Vittimberga, Antonietta Letizia, Andrea Misino, Angela Gernone, Erminio Bonizzoni, Sara Pilotto, Sabino De Placido, Emilio Bria

**Affiliations:** 1 Medical Oncology Unit, ASST del Garda, Località Montecroce 1, 25015 Desenzano del Garda, BS Italy; 2 Medical Oncology Department, ASST Monza San Gerardo Hospital, Monza, Italy; 3 Medical Oncology Department, Azienda Ospedaliero-Universitaria di Sassari, Sassari, Italy; 4 Oncology Department, Azienda Ospedaliera di Piacenza, Piacenza, Italy; 5 Medical Oncology, ASL Frosinone, Frosinone, Italy; 6 Department of Oncology, San Luigi Gonzaga Hospital, University of Turin, Orbassano, Turin, Italy; 7 Cancer Centre Department – Oncology Unit, ASST Ovest Milanese – Legnano Hospital, Legnano, Milan Italy; 8 Department of Oncology and Hematology, AOU Policlinico di Modena, Modena, Italy; 9 Medical Oncology Unit, ASST Bergamo Ovest, Treviglio, Bergamo, Italy; 10 Comprehensive Cancer Center, Fondazione Policlinico Universitario Agostino Gemelli, IRCCS, Rome, Italy; 11 Medical Oncology, Università Cattolica del Sacro Cuore, Rome, Italy; 12 Oncology Unit, AULSS6 Euganea, Padova, Italy; 13 Medical Oncology Unit, IRCCS Santa Maria Nuova, Reggio Emilia, Italy; 14 Medical Oncology Department, San Giuseppe Moscati Hospital, Statte, Taranto, Italy; 15 Department of Oncology, ASST Lecco, Lecco, Italy; 16 Department of Pneumology and Oncology, AORN dei Colli-Ospedale Monaldi, Naples, Italy; 17 Medical Oncology, Clinical Cancer Center, “Giovanni Paolo II” – IRCCS, Bari, Italy; 18 Medical Oncology Unit, University of Bari, Policlinico di Bari, Bari, Italy; 19 Department of Clinical Science and Community, Section of Medical Statistics, Biometry and Epidemiology “G.A. Maccacaro”, Faculty of Medicine and Surgery, University of Milan, Milan, Italy; 20 Section of Oncology, Department of Medicine, University and Hospital Trust of Verona, Verona, Italy; 21 Clinical Medicine and Surgery Department, University of Naples “Federico II”, Naples, Italy

## Abstract

We demonstrated the non-inferiority of a dexamethasone (DEX)-sparing (single-dose) regimen with NEPA, a netupitant/palonosetron fixed combination, for preventing chemotherapy-induced nausea and vomiting (CINV) caused by cisplatin. This pre-planned exploratory analysis assessed the effect of the DEX-sparing regimen on a patient’s food intake. Chemotherapy-naïve patients undergoing cisplatin (≥ 70 mg/m^2^) were given NEPA and DEX (12 mg) on day 1 and randomized to receive either no further DEX (DEX1), or oral DEX (4 mg BID) on days 2–4 (DEX4). Patient-reported endpoint maintenance of usual daily food intake was assessed during the 5-days post-chemotherapy. The relationship between usual daily food intake and CINV control, pre-chemotherapy self-rated food intake and BMI-adjusted weight loss (WL) were evaluated. One-hundred fifty-two patients (76/group) were assessable. The proportion of patients reporting maintenance of usual daily food intake was similar in both groups: 69.7% (95% CI, 58.6–78.9) for DEX1 vs. 72.4% (95% CI, 61.4–81.2) for DEX4. Only CINV control was significantly associated with maintenance of usual daily food intake (*P* ≤ 0.001) during the overall phase. The DEX-sparing regimen does not adversely affect patient-reported daily food intake post-chemotherapy. The current analysis adds further insights into antiemetic efficacy of DEX sparing beyond day 1 in the challenging setting of cisplatin.

*Trial registration*: The parent study was registered on ClinicalTrials.gov (NCT04201769).

## Introduction

Prevention of chemotherapy-induced nausea and vomiting (CINV) occurring in the acute (within 24 h of chemotherapy administration) and delayed (day 2 through 5 after chemotherapy administration) phases remains a priority in the oncology setting^1,2^. Patient quality of life (QoL) as well as treatment compliance may be adversely affected when uncontrolled CINV occur. Suboptimal control of CINV has been consistently reported to be associated with an adverse impact on dietary intake that can result in malnutrition^3^. Direct and indirect effects of nausea and vomiting related to cancer chemotherapy make patients susceptible to malnutrition during planned treatment^4^. Currently, a guideline-consistent prophylaxis allows CINV to be controlled in the majority of patients undergoing chemotherapy^5,6^. A triple combination of a neurokinin-1 receptor antagonist (NK-1RA), 5-hydroxytryptamine-3 (5-HT_3_) RA and multiple-day dexamethasone (DEX), with or without olanzapine, is recommended by evidence-based guidelines for prevention of acute and delayed CINV caused by highly emetogenic chemotherapy (HEC) containing cisplatin^1,2^.

Although the antiemetic efficacy of DEX has been long documented in the CINV setting, its administration may induce a range of side-effects, especially when DEX is administered over multiple consecutive days for prevention of delayed CINV in each chemotherapy cycle^7–10^.

Accordingly, clinicians should keep in mind the increased risk of potential side effects due to prophylactic DEX^11^. The DEX-sparing strategy implemented as the second-generation 5-HT_3_RA, palonosetron in combination with single-dose DEX, with or without an NK-1RA, was shown to be as effective as the regimen including additional DEX doses in breast cancer patients undergoing the high-emetic-risk combination of an anthracycline and cyclophosphamide (AC)^12–14^. Also, a randomized, double-blind study demonstrated that DEX administered only on day 1 before chemotherapy initiation was non-inferior to DEX given for three days in the prevention of CINV caused by HEC regimens (AC or cisplatin), when combined with palonosetron and an NK-1RA^15^. However, in this study 77% of patients were women with breast cancer treated with AC, and post-hoc subgroup analyses failed to show the non-inferiority of the DEX-sparing regimen in patients receiving cisplatin^16^. More recently, we demonstrated that efficacy of two different DEX-sparing regimens (i.e., DEX on day 1 only and DEX on days 1–3 after chemotherapy initiation), when administered with NEPA, a fixed-dose combination of netupitant and palonosetron, was non-inferior to the guideline-recommended regimen of DEX on days 1–4 (also with NEPA) in patients undergoing high-dose cisplatin^17^. All eligible patients were also asked whether or not they had usual food intake each day on days 1 to 5 after chemotherapy administration. Although the function of DEX in preventing and treating appetite loss in cancer patients is well-known, there is a lack of data assessing DEX on day 1 only for CINV control on patients’ food intake^18^. Indeed, DEX sparing on days 2 to 4 after cisplatin administration can be expected to adversely affect patient food intake in the days following chemotherapy administration. The main objective of this pre-planned exploratory analysis from the parent study was to assess the self-reported maintenance of usual daily food intake during the 5-day overall study period following cisplatin in patients receiving either the 1-day DEX regimen or the guideline-recommended 4-day DEX regimen. In addition, we sought to explore the relationship between maintenance of usual food intake and factors potentially associated with patient food intake, including pre-chemotherapy patient self-rated food intake, involuntary weight loss (WL), and symptoms common with cancer as well as CINV control. Since the focus of the analysis was to explore whether single-dose DEX adversely affected maintenance of usual daily food intake, only the results of the study arm including single-dose DEX were analyzed in this paper.

## Participants and methods

### Study design

This is a pre-planned exploratory analysis of a phase IIIb, open-label, multicenter, randomized, three-arm study aimed to evaluate the non-inferiority of two DEX-sparing regimens when combined with oral NEPA versus the guideline-consistent DEX regimen in patients receiving cisplatin-containing chemotherapy^17^. The study was done in compliance with the Declaration of Helsinki and the study protocol was approved by the institutional review boards and the Ethics Committees at the coordinating center (*Comitato Etico per la Sperimentazione Clinica delle province di Verona e Rovigo*) and each participating institution. All patients provided written, informed consent. The parent study was registered on the European Union Clinical Trials Register (EudraCT number 2015-005704-29) and on ClinicalTrials.gov (NCT04201769, registered on 17/12/2019).

### Study population and treatment

Eligible patients were ≥ 18 years of age with a confirmed diagnosis of non-small cell lung cancer (NSCLC), chemotherapy-naive and scheduled to receive the first course of cisplatin (≥ 70 mg/m^2^)-based chemotherapy for early, locally advanced or metastatic cancer. In addition, patients were required to have a body mass index (BMI) of at least 18.5 and have no gastrointestinal obstruction or active peptic ulcer. Major exclusion criteria included patients with symptomatic brain metastases, routine use of corticosteroids, or contraindications for corticosteroid use, and patients who were scheduled to receive either concurrent chemo-radiation therapy or radiation therapy to the abdomen or pelvis within 1 week before chemotherapy initiation. Full eligibility criteria for the study were reported elsewhere^17^. In the parent study, random assignment (1:1:1 ratio) was centrally done using a computer-generated, allocation list. In the DEX-sparing group, patients were given NEPA and DEX (12 mg) on day 1 and no further prophylaxis (DEX1), while, in the reference group, patients received the same prophylaxis on day 1 and additional oral DEX doses (4 mg twice daily) on days 2–4 (DEX4). Patients were allowed to take rescue medication (DEX or metoclopramide) throughout the study period for nausea or vomiting, if necessary.

### Data collection

During days 1 to 5 after chemotherapy administration, patients recorded the following items in their symptom diary every 24 h: the number of emetic episodes and time of first vomiting; severity of nausea using a Likert scale (0, no nausea; 1, mild nausea; 2, moderate nausea; 3, severe nausea); number of rescue medications and time of the first administration. Patients were also asked to record in their diary whether or not they had usual food intake each day on days 1 to 5. In the screening phase, patient’s weight, height, and BMI [weight (kg)/height (m^2^)] were recorded by the treating physician. Patient-reported involuntary WL history over the preceding 6 months was also collected. From this, %WL was calculated as follows: [(current weight in kg – previous weight in kg)/previous weight in kg] × 100^19^. WL grade was assessed and given a score of 0–4 by combining %WL and current BMI according to the WL grading system (WLGS), a validated grading system (grade 0 to 4) for cancer-associated WL based on risk stratification with survival as the outcome^19^. A basic assessment of food intake was carried out using a self-reported question regarding amount of food intake in the previous month adapted from the Patient-Generated Subjective Global Assessment, a validated nutrition assessment tool in cancer patients^20^. In the screening phase, as compared to their normal intake, patients self-rated food intake during the past month as unchanged, more than usual, less than usual, and very less than usual. Patients also recorded pre-chemotherapy experience of cancer-related symptoms by the Edmonton Symptom Assessment System (ESAS), a widely used, self-report symptom intensity tool for assessing 9 common symptoms in cancer patients, with an 11-point numerical rating scale (NRS) ranging from 0 (none, best) to 10 (worst possible)^21^. The ESAS tool was translated into Italian and validated both linguistically and psychometrically^22^. The presence of a symptom was defined a priori as a score of 1 or greater, and clinically significant symptom intensity was defined as a score of 3 or greater^23^. The primary efficacy endpoint of the parent study was the proportion of patients experiencing complete response (CR; defined as no emetic episode and no use of rescue medication) in the overall study period (day 1 through 5 post-chemotherapy). The proportion of patients with no significant nausea (NSN; defined as none or only mild nausea) was a secondary efficacy endpoint. The patient-reported endpoint for the present analysis was proportion of patients reporting usual daily food intake in each treatment group during the overall period. The proportion of patients who reported usual food intake during the acute (0–24 h post-chemotherapy) and delayed (day 2 through 5 post-chemotherapy) phases were also assessed.

### Statistical analysis

For all analyses, we used the per-protocol cohort which comprised all patients who completed study and who were compliant with the study protocol^17^. Data were expressed as mean values (with standard deviation; SD) for continuous variables and frequencies (with percentage) for categorical variables. Comparisons between treatment groups were assessed using Fisher’s exact test or chi-square test for categorical variables, and Mann–Whitney *U*-test for skewed data to test for between-group differences in continuous variables. The relationship between pre- and post-chemotherapy factors potentially associated with patient food intake and the maintenance of usual daily food intake (yes vs. no) during the overall study period was assessed by the Mantel–Haenszel chi-square test stratified by antiemetic regimen. A further sensitivity analysis stratified by treatment groups. Indeed, the concordance between pooled and stratified analyses should rule out any randomization bias. Results were reported as odds ratios (ORs) with associated 95% confidence intervals (CIs). All *p* values were two-tailed, and a *P* < 0.05 was considered statistically significant.

## Results

A total of 152 patients were assessed with characteristics by treatment group presented in Table 1. There were no relevant differences between the treatment groups with respect to all baseline demographic, clinical and anthropometric data. Similar rates of patients in each treatment group (63.2% vs. 55.3% in the DEX1 and DEX4 groups, respectively) self-rated their food intake during the past month before the study entry as unchanged. In terms of pre-chemotherapy BMI-adjusted WL, 52 of 76 (68.4%) in the DEX1 group and 47 of 76 (61.8%) patients in the DEX4 group had low-grade WL (grade 0–1).

**Table 1:** Baseline demographic, clinical and anthropometric characteristics of the study patients.

Variables	NEPA + DEX1 (n = 76)	NEPA + DEX4 (n = 76)	*P*-value*
Age (years), mean (SD)	64.4 (7.2)	63.6 (7.9)	0.45
Female sex, n (%)	21 (27.6)	22 (28.9)	1.00
Weight (Kg), mean (SD)	70.2 (13.5)	72.5 (12.7)	0.27
BMI (kg/m^2^), mean (SD)	24.7 (4.1)	25.4 (3.9)	0.28
Healthy weight (18.5–24.9 kg/m^2^), n (%)	43 (56.6)	37 (48.7)	0.69
Overweight (25–29.9 kg/m^2^), n (%)	26 (34.2)	30 (39.5)	
Obese (≥ 30 kg/m^2^), n (%)	7 (9.2)	9 (11.8)	
ECOG PS, n (%)			0.22
0	63 (82.9)	58 (76.3)	
1	13 (17.1)	18 (23.7)	
Tumor stage, n (%)			0.34
Early	20 (26.3)	16 (21.1)	
Locally advanced	27 (35.5)	22 (28.9)	
Metastatic	29 (38.2)	38 (50)	
Alcohol intake, n (%)			0.48
Never	55 (72.4)	50 (65.8)**	
Everyday	21 (27.6)	26 (34.2)	
Percentage WL in previous 6 months			
Mean (SD)	− 1.6 (5.5)	− 2.3 (5.1)	0.39
***Pre-chemotherapy food intake, n (%)			0.50
More than usual	7 (9.2)	11 (14.4)	
Unchanged	48 (63.2)	42 (55.3)	
Less than usual	21 (27.6)	23 (30.3)	
BMI-adjusted WL grade, n (%)			0.17
0	29 (38.2)	28 (36.8)	
1	23 (30.3)	19 (25)	
2	4 (5.2)	14 (18.4)	
3	15 (19.7)	10 (13.2)	
4	5 (6.6)	5 (6.6)	

*NEPA* fixed combination of netupitant and palonosetron, *DEX1* dexamethasone day 1, *DEX4* dexamethasone day 1 to 4, *SD* standard deviation, *BMI* body mass index, *ECOG PS* Eastern Cooperative Oncology Group performance status, *WL* weight loss.

*p-value was calculated using Fisher’s exact test, chi-square test, and Mann–Whitney U-test as appropriate; all tests were two-tailed.

**Including a patient with missing data.

***As compared with their normal food intake, patients self-rated food intake during the past month before the study entry.

The prevalence of pre-chemotherapy cancer-related symptoms (score of ≥ 1) assessed by ESAS was similar between treatment groups, with no significant differences observed (Table 2). Likewise, the frequency of clinically significant symptom intensities (score of ≥ 3) was similar between the groups.

**Table 2:** Descriptive summary of pre-chemotherapy cancer-related symptoms by treatment group.

Symptoms	NEPA + DEX1 (n = 76)	NEPA + DEX4 (n = 76)	*p*-value*
Mean score (SD) for pain, (0–10 NRS)	1.25 (2.3)	1.68 (2.6)	0.41
Pain (≥ 1 NRS), n (%)	28 (36.8)	32 (42.1)	0.62
Significant pain (≥ 3 NRS), n (%)	13 (17.1)	18 (23.7)	0.42
Mean score (SD) for tiredness, (0–10 NRS)	2.14 (2.5)	2.11 (2.7)	0.70
Tiredness (≥ 1 NRS), n (%)	45 (59.2)	40 (52.6)	0.51
Significant tiredness (≥ 3 NRS), n (%)	29 (38.2)	24 (31.6)	0.50
Mean score (SD) for nausea, (0–10 NRS)	0.33 (0.9)	0.45 (1.5)	0.98
Nausea (≥ 1 NRS), n (%)	11 (14.5)	11 (14.5)	1.00
Significant nausea (≥ 3 NRS), n (%)	3 (3.9)	5 (6.6)	0.72
Mean score (SD) for depression, (0–10 NRS)	1.09 (1.7)	1.21 (2.2)	0.65
Depression (≥ 1 NRS), n (%)	31 (40.8)	26 (34.2)	0.50
Significant depression (≥ 3 NRS), n (%)	16 (21.1)	14 (18.4)	0.84
Mean score (SD) for anxiety, (0–10 NRS)	1.43 (2.0)	1.86 (2.7)	0.92
Anxiety (≥ 1 NRS), n (%)	39 (51.3)	35 (46.1)	0.63
Significant anxiety (≥ 3 NRS), n (%)	17 (22.4)	22 (28.9)	0.46
Mean score (SD) for drowsiness, (0–10 NRS)	1.76 (2.4)	1.66 (2.5)	0.41
Drowsiness (≥ 1 NRS), n (%)	41 (53.9)	32 (42.1)	0.19
Significant drowsiness (≥ 3 NRS), n (%)	20 (26.3)	20 (26.3)	1.00
Mean score (SD) for loss of appetite loss, (0–10 NRS)	1.36 (2.4)	1.57 (2.5)	0.79
Loss of appetite (≥ 1 NRS), n (%)	30 (39.5)	30 (39.5)	1.00
Significant loss of appetite (≥ 3 NRS), n (%)	14 (18.4)	19 (25)	0.43
Mean score (SD) for well-being, (0–10 NRS)	3.53 (2.7)	3.82 (2.7)	0.43
Poor well-being (≥ 1 NRS), n (%)	63 (82.9)	64 (84.2)	1.00
Significant poor well-being (≥ 3 NRS), n (%)	48 (63.2)	49 (64.5)	1.00
Mean score (SD) for difficulty of breathing, (0–10 NRS)	1.11 (1.7)	1.25 (2.0)	0.88
Difficulty of breathing (≥ 1 NRS), n (%)	32 (42.1)	29 (38.2)	0.74
Significant difficulty of breathing (≥ 3 NRS), n (%)	15 (19.7)	15 (19.7)	1.00

All tests were two-tailed.

*NEPA* fixed combination of netupitant and palonosetron, *DEX1* dexamethasone day 1, *DEX4* dexamethasone day 1 to 4, *SD* standard deviation, *NRS* numerical rating scale (with 10 being the most severe).

*p-value was calculated using Mann–Whitney U-test and Fisher’s exact test as appropriate.

### Usual daily food intake after chemotherapy

**Figure 1:**
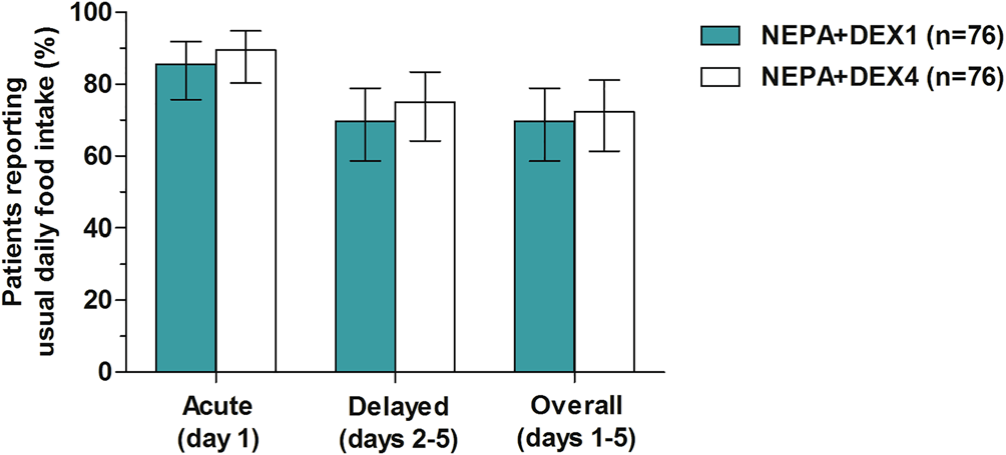
Proportion of patients reporting usual daily food intake in acute, delayed, and overall phases. Error bars represent 95% confidence interval. NEPA, fixed combination of netupitant and palonosetron; DEX1, dexamethasone day 1; DEX4, dexamethasone day 1 to 4.

**Figure 2:**
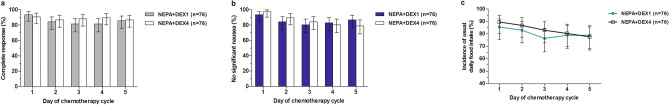
Time course of complete response (**a**), no significant nausea (**b**), and usual daily food intake (**c**) in each treatment group (by 24-h period). Error bars represent 95% confidence interval. NEPA, fixed combination of netupitant and palonosetron; DEX1, dexamethasone day 1; DEX4, dexamethasone day 1 to 4.

The proportion of patients reporting maintenance of usual daily food intake during the overall period after cisplatin administration was similar in both treatment groups: 69.7% (95% CI, 58.6 to 78.9) in the DEX1 group vs. 72.4% (95% CI, 61.4 to 81.2) in the DEX4 group (*P* = 0.86; Fig. 1). Similar rates of usual daily food intake were also observed in the DEX1 group compared with the DEX4 group during the acute (85.5% [95% CI, 75.7 to 91.9] vs. 89.5% [95% CI, 80.3 to 94.8]; *P* = 0.63) and delayed (69.7% vs. 75% [95% CI, 64.2 to 83.4]; *P* = 0.59; Fig. 1) periods. When CINV prevention was analyzed on each of the 5 days during the overall phase, CR and NSN rates on days 2 (CR: 84.2% vs. 86.8%, *P* = 0.82; NSN: 84.2% vs. 89.5%, *P* = 0.47) and 3 (CR: 81.6% vs. 88.2%, *P* = 0.37; NSN: 80.3% vs. 84.2%, *P* = 0.67) were slightly lower in patients receiving the DEX-sparing regimen (Fig. 2a,b), although no statistically significant differences were observed between groups at all daily intervals. Consistent with these findings, the rates of usual daily food intake on days 2 and 3 were also slightly lower in patients receiving the DEX-sparing regimen, although there were no statistically significant between-group differences (day 2: 82.9% vs. 86.8%, *P* = 0.65; day 3: 76.3% vs. 82.9%, *P* = 0.42; Fig. 2c).

### Cancer-related symptoms and maintenance of daily food intake

Table 3 shows the relationship between pre-chemotherapy cancer-related symptoms and maintenance of patient-reported usual daily food intake. In analyses stratified by antiemetic regimen, we did not observe any significant relationship between pain (*P* = 0.13), tiredness (*P* = 0.77), nausea (*P* = 0.28), depression (*P* = 0.98), anxiety (*P* = 0.28), drowsiness (*P* = 0.65), loss of appetite (*P* = 0.13), poor well-being (*P* = 0.72) or difficulty of breathing (*P* = 0.94) and maintenance of usual daily food intake during the overall phase.

**Table 3:** Analysis of the relationship between pre-chemotherapy cancer-related symptoms and maintenance of usual daily food intake in the overall phase (day 1 to 5).

Variables*	Subgroup	No. of patients	NEPA + DEX1 (n = 76)		No. of patients	NEPA + DEX4 (n = 76)		OR (95% CI)	*p*-value**
			Usual daily food intake			Usual daily food intake			
			N	%		N	%		
Pain	Yes	28	18	64.3	32	20	62.5	0.54	0.13
	No	48	35	72.9	44	35	79.5	(0.26; 1.10)	
Tiredness	Yes	45	31	68.9	40	28	70	0.84	0.77
	No	31	22	70.9	36	27	75	(0.41; 1.71)	
Nausea	Yes	11	7	63.6	11	6	54.5	0.53	0.28
	No	65	46	70.8	65	49	75.4	(0.21; 1.36)	
Depression	Yes	31	22	70.9	26	19	73.1	1.08	0.98
	No	45	31	68.9	50	36	72	(0.52; 2.24)	
Anxiety	Yes	39	25	64.1	35	24	68.6	0.63	0.28
	No	37	28	75.7	41	31	75.6	(0.31; 1.29)	
Drowsiness	Yes	41	28	68.3	32	22	68.8	0.80	0.65
	No	35	25	71.4	44	33	75	(0.39; 1.62)	
Loss of appetite	Yes	30	17	56.7	30	21	70	0.55	0.13
	No	46	36	78.3	46	34	73.9	(0.27; 1.11)	
Poor well-being	Yes	63	43	68.3	64	46	71.9	0.74	0.72
	No	13	10	76.9	12	9	75	(0.27; 1.99)	
Difficulty of breathing	Yes	32	21	65.6	29	22	75.9	0.96	0.94
	No	44	32	72.7	47	33	70.2	(0.47; 1.96)	

*NEPA* fixed combination of netupitant and palonosetron, *DEX1* dexamethasone day 1, *DEX4* dexamethasone day 1 to 4, *OR* odds ratio, *CI* confidence interval.

*Self-rated by the patient.

**p-value was calculated using the Mantel–Haenszel chi-square test (two-tailed) stratified by treatment group.

### Patient-related factors and maintenance of daily food intake

Table 4 shows the relationship between pre-chemotherapy patient-related factors and maintenance of patient-reported usual daily food intake. In analyses stratified by antiemetic regimen, we did not observe any significant relationship between age (*P* = 0.83), sex (*P* = 0.67), alcohol intake (*P* = 0.45), performance status (*P* = 0.83), tumor stage (*P* = 0.99), degree of food intake (*P* = 0.27) or BMI-adjusted WL grade (*P* = 0.65) and maintenance of usual daily food intake during the overall phase.

**Table 4:** Analysis of the relationship between pre-chemotherapy patient-related factors and maintenance of usual daily food intake in the overall phase (day 1 to 5).

Variables	Subgroup	No. of patients	NEPA + DEX1 (n = 76)		No. of patients	NEPA + DEX4 (n = 76)		OR (95% CI)	*p*-value*
			Usual daily food intake			Usual daily food intake			
			N	%		N	%		
Age	≥ 55 years	69	48	69.6	66	48	72.7	1.04	0.83
	< 55 years	7	5	71.4	10	7	70	(0.34; 3.15)	
Sex	Female	21	15	71.4	22	14	63.6	0.79	0.67
	Male	55	38	69.1	54	41	75.9	(0.37; 1.68)	
Alcohol intake	No	55	41	74.5	50	36	72	1.43	0.45
	Everyday	21	12	57.1	26	19	73.1	(0.68; 3.0)	
ECOG PS score	0	63	42	66.7	58	43	74.1	0.83	0.86
	1	13	11	84.6	18	12	66.7	(0.34; 2.03)	
Tumor stage	Early	20	15	75	16	10	62.5	0.91	0.99
	Advanced	56	38	67.9	60	45	75	(0.41; 2.05)	
Food intake**	More than usual or Unchanged	55	38	69.1	53	42	79.2	1.63	0.27
	Less than usual	21	15	71.4	23	13	56.5	(0.77; 3.43)	
BMI-adjusted	0–1	52	35	67.3	47	37	78.7	1.27	0.65
WL grade***	2–4	24	18	75	29	18	62.1	(0.61; 2.62)	

*NEPA* fixed combination of netupitant and palonosetron, *DEX1* dexamethasone day 1, *DEX4* dexamethasone day 1 to 4, *OR* odds ratio, *CI* confidence interval, *ECOG PS* Eastern Cooperative Oncology Group performance status, *BMI* body mass index, *WL* weight loss.

*p-value was calculated using the Mantel–Haenszel chi-square test (two-tailed) stratified by treatment group.

**As compared with their normal food intake, patients self-reported food intake during the past month before the study entry.

***Increasing WL grades are associated with reduced quality of life and reduced survival.

### CINV control and maintenance of daily food intake

Table 5 shows the relationship between CINV control assessed by CR and NSN and maintenance of patient-reported usual daily food intake. Similar rates of patients in each treatment group who experienced a CR or NSN reported usual daily food intake during the overall phase. In analyses stratified by antiemetic regimen, there was a significant relationship between CINV control and maintenance of usual daily food intake (*P* ≤ 0.001).

**Table 5:** Analysis of the relationship between CINV control and maintenance of usual daily food intake in the overall phase (day 1 to 5).

Variables	Subgroup	No. of patients	NEPA + DEX1 (n = 76)		No. of patients	NEPA + DEX4 (n = 76)		OR (95% CI)	*p*-value*
			Usual daily food intake			Usual daily food intake			
			N	%		N	%		
CR (overall phase)	Yes	58	45	77.6	57	45	78.9	3.82	0.001
	No	18	8	44.4	19	10	52.6	(1.74; 8.35)	
NSN (overall phase)	Yes	59	46	77.9	58	46	79.3	4.39	0.0004
	No	17	7	41.2	18	9	50	(1.97; 9.77)	

*NEPA* fixed combination of netupitant and palonosetron, *DEX1* dexamethasone day 1, *DEX4* dexamethasone day 1 to 4, *OR* odds ratio, *CI* confidence interval, *CR* complete response (no vomiting and no rescue medication), *NSN* no significant nausea (none or mild nausea).

*p-value was calculated using the Mantel–Haenszel chi-square test (two-tailed) stratified by treatment group.

## Discussion

Nausea and vomiting are considered nutrition-influencing symptoms, and patients who experience CINV are particularly susceptible to malnutrition which in turn causes impairments in performance status and QoL^4^. Also, it is known that DEX has appetite-stimulating efficacy in cancer patients^18^. Therefore, it is important to assess whether reducing patient’s exposure to DEX will compromise not only the ability to effectively control CINV but also daily food intake. We recently demonstrated the non-inferiority of DEX sparing on days 2 to 4, combined with NEPA, compared with a guideline-consistent use of DEX for CR during the overall phase of CINV in NSCLC patients receiving high-dose cisplatin^17^. The current analysis showed no detrimental effect of single-dose DEX on patient-reported usual daily food intake during the 5-day observation period of CINV. Approximately 70% of patients in both the DEX1 and DEX4 groups reported maintenance of usual daily food intake during the overall study period following administration of cisplatin in cycle 1. Similar rates of usual daily food intake were also observed between groups during the acute and delayed study periods. In analyses stratified by antiemetic regimen including a number of pre- and post-chemotherapy factors potentially associated with patient food intake, there was only a strongly significant relationship between CINV control and patient-reported maintenance of usual daily food intake during the overall period. No significant relationship was observed between pre-chemotherapy common cancer-related symptoms, self-rated degree of food intake, or BMI-adjusted WL grade and maintenance of usual daily food intake. It should be noted that most patients who were fit to receive high-dose cisplatin (≥ 70 mg/m^2^) had both an unchanged pre-chemotherapy food intake and low-grade WL which could explain the lack of relationship between either variable and maintenance of usual daily food intake. The BMI-adjusted WLGS is a grading system for cancer-associated WL based on risk stratification with survival as the outcome and these observations are independent of tumor site, stage, and performance status^19^. WL grade is also associated with cachexia-related domains^24^. Of these, dietary intake and loss of appetite are the factors most strongly related to increasing WL grade, with the risk of cachexia progression being considerably higher in WL grade 2 compared with that in WL grade 0 or 1^24^.

A recent prospective study of NSCLC patients undergoing cisplatin-based HEC identified loss of appetite as one of the five most-frequent symptoms reported by patients within the chemotherapy cycle^25^. Also, loss of appetite was at a moderate level on days 3 to 7 post-chemotherapy, and then reduced to a stable and low level in the following two weeks^25^. This supports the assessment of food intake at five time points during the overall period after cisplatin administration, focusing on the time period when risk of CINV is highest. When the daily rates of patient-reported usual food intake were compared, they decreased from day 1 to day 3 in the DEX1 group and from days 1–5 in the DEX4 group; however, no significant differences were observed between treatment groups on any of the individual days 1–5. The decrease was numerically greater in the DEX1 group on days 2 and 3 when a similar decrease also occurred for CR and NSN rates. These findings are consistent with literature that reports the highest incidence of delayed CINV, especially nausea, occurs during the period from 48 to 72 h after administration of cisplatin^26^. It also should be noted that in the study by Ito et al.^15^ where 77% of patients received AC instead of cisplatin, the proportion of patients reporting NSN was at its lowest value on day 3 in the DEX-sparing group. Interestingly, this study also showed that loss of appetite on days 2 and 3 was more frequently reported in the DEX-sparing group than in the reference group^15^. It is well-known that loss of appetite in cancer patients undergoing chemotherapy interlinks with nausea^27^. While in our study no significant differences were seen between the DEX1 group and the DEX4 group, it is worth noting that DEX administration on days 2 and 3 in the DEX4 group might have lessened the severity of nausea in some patients and consequently alleviated both cisplatin-related loss of appetite and impairment in daily food intake to some extent. This view is supported by the following observations: (a) the relationship between CINV control and patient-reported maintenance of usual daily food intake was stronger when assessing NSN, an endpoint which evaluates the proportion of patients free of moderate-to-severe nausea, (b) the degree of nausea control is more likely to influence self-rated appetite and food liking^28^, and (c) in a recently published analysis of health-related QoL using the validated tool of the Functional Living Index-Emesis (FLIE), the mean FLIE scores for vomiting domain during the overall phase in the DEX-sparing groups were comparable to that in the DEX4 group, while the mean FLIE scores for nausea domain were slightly lower in both DEX-sparing groups^29^. Although the parent study did not show a statistically significant difference between the DEX-sparing regimens vs. DEX4 for the secondary efficacy endpoint of no nausea during both the delayed and overall periods^17^, the absolute values suggest that clinicians should be aware that there is room for further improving control of nausea by adding olanzapine. Interestingly, a recent phase III trial found that nausea control was significantly improved by adding low-dose olanzapine to a triple regimen containing palonosetron, aprepitant, and multiple-day DEX in patients receiving cisplatin^30^. Therefore, the addition of olanzapine should be considered in select patients who receive DEX-sparing regimens when nausea control may be an issue.

There are several limitations in this study. First, this is a pre-planned exploratory analysis of the parent study, and the findings should be considered preliminary. Second, the current analysis was not designed as a nutrition trial. Since more detailed data about patients’ nutrition status were not collected, we did not account for their possible impact on our findings. Third, the qualitative data from the patient’s diary reflect only whether maintenance of daily food intake occurs or not but not the severity of the impact on food intake and its variation tendency during chemotherapy. Finally, the study assessment was not done in consecutive cycles of therapy since antiemetic efficacy could be evaluated only over cycle 1 in an investigator-initiated study. It is known that control of acute and delayed CINV in the prior cycle of treatment can influence the occurrence of delayed CINV in the subsequent cycle^31^. Since impairment in food intake can be detrimental to patients, especially for those with metastatic cancer, future research should focus on how daily food intake in patients receiving a DEX-sparing antiemetic regimen changes over consecutive cycles of cisplatin. Despite these limitations, in the absence of data on the impact of a DEX-sparing regimen on patients’ food intake, the prospective and randomized nature of the current analysis offers preliminary but valuable insights.

## Conclusion

The current analysis suggests that, despite DEX sparing beyond day 1, patients who received high-dose cisplatin did not experience any adverse impact on their usual daily food intake (as self-reported by the patient) during the overall phase of CINV. Also, patients experiencing a CR or NSN were more likely to report maintenance of usual daily food intake regardless of DEX regimen administered. Overall, the current analysis adds further insights into the efficacy of the DEX-sparing regimen with NEPA in the challenging setting of CINV caused by cisplatin.

## Data Availability

The datasets analyzed during the present study will be available on reasonable request. The corresponding author should be contacted to request the data.

## References

[1] Roila F (2016). 2016 MASCC and ESMO guideline update for the prevention of chemotherapy- and radiotherapy-induced nausea and vomiting and of nausea and vomiting in advanced cancer patients. Ann. Oncol..

[2] Hesketh PJ (2017). Antiemetics: American Society of Clinical Oncology clinical practice guideline update. J. Clin. Oncol..

[3] Farrell C, Brearley SG, Pilling M, Molassiotis A (2013). The impact of chemotherapy-related nausea on patients’ nutritional status, psychological distress and quality of life. Support. Care Cancer.

[4] Marx W, Kiss N, McCarthy AL, McKavanagh D, Insering L (2016). Chemotherapy-induced nausea and vomiting: A narrative review to inform dietetics practice. J. Acad. Nutr. Diet.

[5] Aapro M (2012). The effect of guideline-consistent antiemetic therapy on chemotherapy-induced nausea and vomiting (CINV): The Pan European Emesis Registry (PEER). Ann. Oncol..

[6] Gilmore JW (2014). Antiemetic guideline consistency and incidence of chemotherapy-induced nausea and vomiting in US community oncology practice: INSPIRE study. J. Oncol. Pract..

[7] Vardy J, Chiew KS, Galica J, Pond GR, Tannock IF (2006). Side effects associated with the use of dexamethasone for prophylaxis of delayed emesis after moderately emetogenic chemotherapy. Br. J. Cancer.

[8] Han HS (2015). A prospective multicenter study evaluating secondary adrenal suppression after antiemetic dexamethasone therapy in cancer patients receiving chemotherapy: A Korean South West Oncology Group study. Oncologist.

[9] Nakamura M (2017). A prospective observational study on effect of short-term periodic steroid premedication on bone metabolism in gastrointestinal cancer (ESPRESSO-01). Oncologist.

[10] Jeong Y (2016). A pilot study evaluating steroid-induced diabetes after antiemetic dexamethasone therapy in chemotherapy-treated cancer patients. Cancer Res. Treat..

[11] Jordan K, Jahn F, Aapro M (2015). Recent developments in the prevention of chemotherapy-induced nausea and vomiting (CINV): A comprehensive review. Ann. Oncol..

[12] Aapro M (2010). Double-blind, randomised, controlled study of the efficacy and tolerability of palonosetron plus dexamethasone for 1 day with or without dexamethasone on days 2 and 3 in the prevention of nausea and vomiting induced by moderately emetogenic chemotherapy. Ann. Oncol..

[13] Aapro M (2014). A randomized phase III study evaluating the efficacy and safety of NEPA, a fixed-dose combination of netupitant and palonosetron, for prevention of chemotherapy-induced nausea and vomiting following moderately emetogenic chemotherapy. Ann. Oncol..

[14] Celio L (2019). Impact of dexamethasone-sparing regimens on delayed nausea caused by moderately or highly emetogenic chemotherapy: A meta-analysis of randomised evidence. BMC Cancer.

[15] Ito Y (2018). Placebo-controlled, double-blind phase III study comparing dexamethasone on day 1 with dexamethasone on days 1 to 3 with combined neurokinin-1 receptor antagonist and palonosetron in high-emetogenic chemotherapy. J. Clin. Oncol..

[16] Celio L, Bonizzoni E, Aapro M (2018). Is the dexamethasone-sparing strategy ready for cisplatin? Too early for an answer. J. Clin. Oncol..

[17] Celio L (2021). Dexamethasone-sparing regimens with oral netupitant and palonosetron for the prevention of emesis caused by high-dose cisplatin: A randomized noninferiority study. Oncologist.

[18] Chang VT, Ingham J (2003). Symptom control. Cancer Invest..

[19] Martin L (2015). Diagnostic criteria for the classification of cancer-associated weight loss. J. Clin. Oncol..

[20] Bauer J, Capra S, Ferguson M (2002). Use of the scored Patient-Generated Subjective Global Assessment (PG-SGA) as a nutrition assessment tool in patients with cancer. Eur. J. Clin. Nutr..

[21] Watanabe SM (2011). A multicentre comparison of two numerical versions of the Edmonton Symptom Assessment System in palliative care patients. J. Pain Symptom Manag..

[22] Moro C (2006). Edmonton symptom assessment scale: Italian version in two palliative care settings. Support. Care Cancer.

[23] Caputo R (2020). Netupitant/palonosetron (NEPA) and dexamethasone for prevention of emesis in breast cancer patients receiving adjuvant anthracycline plus cyclophosphamide: A multi-cycle, phase II study. BMC Cancer.

[24] Vagnildhaug OM (2017). The applicability of a weight loss grading system in cancer cachexia: A longitudinal analysis. J. Cachexia Sarcopenia Muscle.

[25] Liu J (2021). Symptom trajectories during chemotherapy in patients with non-small cell lung cancer (NSCLC) and the function of prolonging low dose dexamethasone in promoting enhanced recovery after chemotherapy. Thorac. Cancer.

[26] Kris MG (1985). Incidence, course, and severity of delayed nausea and vomiting following the administration of high-dose cisplatin. J. Clin. Oncol..

[27] Molassiotis A, Farrell C, Bourne K, Brearley SG, Pilling M (2012). An exploratory study to clarify the cluster of symptoms predictive of chemotherapy-related nausea using random forest modeling. J. Pain Symptom Manag..

[28] Boltong A (2014). A prospective cohort study of the effects of adjuvant breast cancer chemotherapy on taste function, food liking, appetite and associated nutritional outcomes. PLoS ONE.

[29] Celio L (2022). Evaluating the impact of chemotherapy-induced nausea and vomiting on daily functioning in patients receiving dexamethasone-sparing antiemetic regimens with NEPA (netupitant/palonosetron) in the cisplatin setting: Results from a randomized phase 3 study. BMC Cancer.

[30] Hashimoto H (2020). Olanzapine 5 mg plus standard antiemetic therapy for the prevention of chemotherapy-induced nausea and vomiting (J-FORCE): A multicentre, randomised, double-blind, placebo-controlled, phase 3 trial. Lancet Oncol..

[31] Italian Group for Antiemetic Research (1994). Cisplatin-induced delayed emesis: Pattern and prognostic factors during three subsequent cycles. Ann. Oncol..

